# Animal Learning in a Multidimensional Discrimination Task as Explained by Dimension-Specific Allocation of Attention

**DOI:** 10.3389/fnins.2018.00356

**Published:** 2018-06-05

**Authors:** Flavia Aluisi, Anna Rubinchik, Genela Morris

**Affiliations:** ^1^Sagol Department of Neurobiology, University of Haifa, Haifa, Israel; ^2^Department of Economics, University of Haifa, Haifa, Israel

**Keywords:** reinforcement learning, attention, set-shifting, rule-learning, animal behavior

## Abstract

Reinforcement learning describes the process by which during a series of trial-and-error attempts, actions that culminate in reward are reinforced, becoming more likely to be chosen in similar circumstances. When decisions are based on sensory stimuli, an association is formed between the stimulus, the action and the reward. Computational, behavioral and neurobiological accounts of this process successfully explain simple learning of stimuli that differ in one aspect, or along a single stimulus dimension. However, when stimuli may vary across several dimensions, identifying which features are relevant for the reward is not trivial, and the underlying cognitive process is poorly understood. To study this we adapted an intra-dimensional/ extra-dimensional set-shifting paradigm to train rats on a multi-sensory discrimination task. In our setup, stimuli of different modalities (spatial, olfactory and visual) are combined into complex cues and manipulated independently. In each set, only a single stimulus dimension is relevant for reward. To distinguish between learning and decision-making we suggest a weighted attention model (WAM). Our model learns by assigning a separate learning rule for the values of features of each dimension (e.g., for each color), reinforced after every experience. Decisions are made by comparing weighted averages of the learnt values, factored by dimension specific weights. Based on the observed behavior of the rats we estimated the parameters of the WAM and demonstrated that it outperforms an alternative model, in which a learnt value is assigned to each combination of features. Estimated decision weights of the WAM reveal an experience-based bias in learning. In the first experimental set the weights associated with all dimensions were similar. The extra-dimensional shift rendered this dimension irrelevant. However, its decision weight remained high for the early learning stage in this last set, providing an explanation for the poor performance of the animals. Thus, estimated weights can be viewed as a possible way to quantify the experience-based bias.

## 1. Introduction

A common conceptualization of choices between alternatives highlights two components to the decision process: outcome valuation and mapping of states and actions to outcomes (Rangel et al., 2008; Gilboa and Marinacci, 2016). Even if the subjective value of each outcome is known, computing similarity between different states, as required for state-action-outcome mapping, is a hard problem (Erev and Roth, 2014; Argenziano and Gilboa, 2017). Lack of knowledge about the environment might either lead to avoiding unknown environments, as in Ellsberg (1961), or may motivate exploration and learning (Rangel et al., 2008). Modeling approaches of the learning process in decision making contexts have differed between fields of research. Whereas in economics it has been predominantly based on the Bayesian framework (Gilboa and Marinacci, 2016), a frequentist-based reinforcement learning (RL) (Sutton and Barto, 1998) approach was instrumental in explaining human and animal choice behavior and in pointing to the function of the underlying neurobiological structures.

Classical RL models (Wagner and Rescorla, 1972; Sutton and Barto, 1998) assume that a decision-maker assigns a value to each action (or action-state pair) and updates it according to the reward history. These values form the basis for decisions. However, there is clearly more to adaptive choice behavior than simple updating of values. As similar problems are encountered, learning is significantly facilitated. This implies a higher level of meta-learning, in which the learning process itself is improved. Meta-learning can take many forms, from tuning of parameters (Doya, 2002) to extracting underlying patterns (Plonsky et al., 2015) or problem characteristics (Gershman and Niv, 2010; Collins and Frank, 2013). Rather than adopting individual solutions, a rule is derived for the family of problems, forming a learning set (Harlow, 1949). Learning sets require uncovering the underlying structure of the task set, allowing generalization. Learning in such scenarios involves two stages: structure learning and parametric learning (Braun et al., 2010).

We will focus here on category learning, where categories are naturally defined by types of sensory input, only a subset of which are relevant to the outcome (Mackintosh and Little, 1969; Roberts et al., 1988). In this common case, it is beneficial to extract only the relevant aspects of the high-dimensional input to simplify learning and decision-making. This may be achieved in humans by introducing selective attention to different types of information in the learning process (Slamecka, 1968; Niv et al., 2015), in the decision process, or both (Leong et al., 2017). Rodent studies in which the rules governing reward involve different dimensions of presented stimuli show that, when appropriately trained, the animals' behavior is consistent with dimension-specific attention sets (Crofts et al., 2001; Chase et al., 2012; Lindgren et al., 2013; Bissonette and Roesch, 2015; Wright et al., 2015; Aoki et al., 2017). However, it is still not clear how these sets are formed, and whether they are learned through reinforcement. To study how animals deal with extraction of a subset of relevant stimulus dimensions in a high-dimensional setting, we adapted an intra-dimensional/extra-dimensional set shifting paradigm to train rodents on a deterministic multidimensional sensory discrimination task involving spatial, olfactory and visual associations. In each set, only a single stimulus dimension is relevant for reward. To account for the animals' behavior, we applied a modified reinforcement learning model, combined with a decision rule that chooses among alternatives by comparing the weighted averages of the corresponding learnt values, factored by dimension-specific weights. We applied the model to the trial-by-trial choice behavior during the various stages of task performance and show that this model out-performs a simple RL model in describing the data, and that it uncovers patterns of animal behavior that cannot be explained by reinforcement statistics alone.

## 2. Results

### 2.1. Behavior

**Figure 1:**
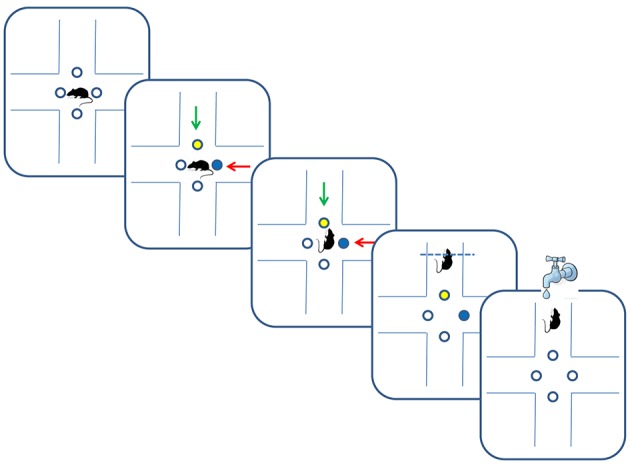
Multidimensional learning paradigm and trial progression. Blue and yellow dots depict illuminated LEDs, and green and red arrows indicate different odors. In the depicted trial, rats received reward for following the green odor in an odor-set phase of the task, or for following the yellow color in a LED-set phase of the task.

The data was collected from 18 Long-Evans rats trained on two versions of a multidimensional sensory discrimination task (Figure 1). In this task, naive water-restricted rats are introduced to a plus-shaped maze. In each trial, the animals choose between two (out of four) randomly chosen arms, marked by light emitting diodes (LEDs) in two colors and two odors. The pair of odors and the pair of colored LEDs were randomly assigned to the two arms. For the duration of each training set, only one sensory dimension (olfactory, visual, or spatial) determined the correct choice. Correct responses were rewarded by a drop of water provided in a port located at the end of the appropriate arm. Training consisted of 50–100 daily trials. We set the threshold of 75% of correct choices during a day as a criterion of successful learning of a given set. After successful learning, we performed an intra-dimensional and/or an extra-dimensional shift. In an intra-dimensional shift, the sensory dimension that determined reward remained the same, but a new association with reward had to be learned. In an extra-dimensional shift, the dimension that previously determined reward was no longer relevant, and the animals had to learn a rule that relied on a different dimension.

**Figure 2:**
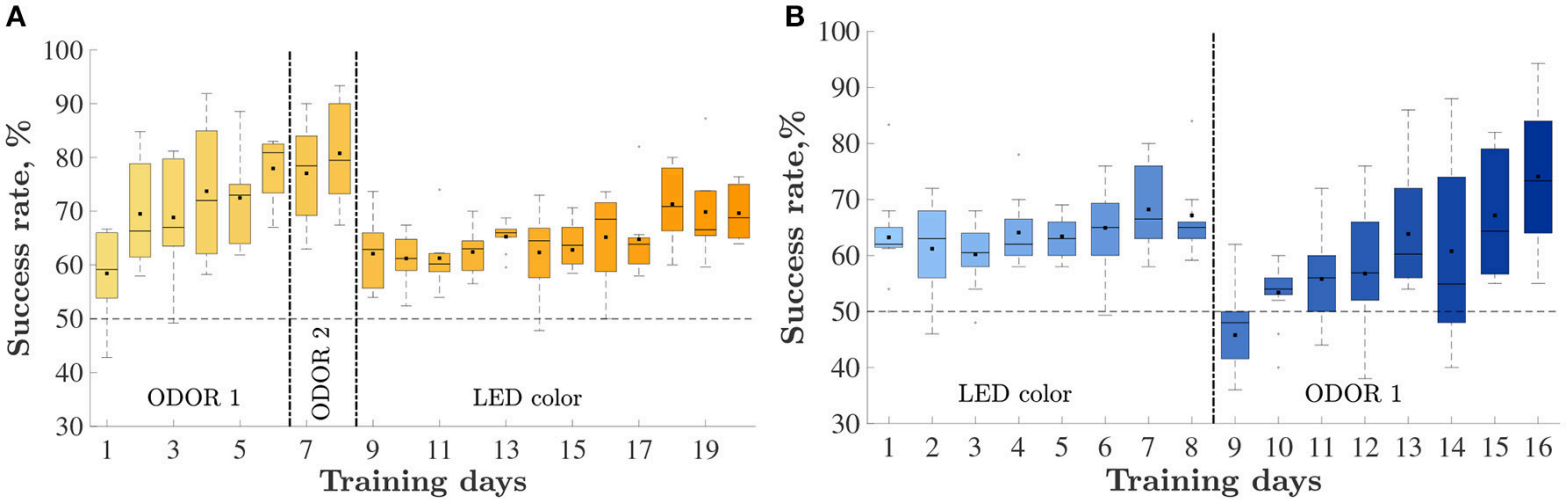
Animals' performance throughout training: percent of correctly performed trials, by day. Vertical lines depict set shifts. The identities of the set in each phase is indicated below. The horizontal line depicts chance level performance. Only days with at least half of the rats are presented. Mean and median success rate in each day are depicted by a black square and horizontal line, respectively. Bars represent the 25 and 75th percentiles correspondingly. **(A)** The odor-first group, *n* = 8. **(B)** The LED-first group, *n* = 10.

Eight animals were trained on an odor-first version of the task (Figure 2A) and 10 were trained on the LED-first version (Figure 2B). In the odor-first group (Figure 2A), during the first set ODOR 1, the animals reached the 75% success rate within 4.3 ± 0.7 days (*mean* ± *S*.*E*.*M*). We subsequently performed an intra-dimensional shift, replacing the pair of odors used for discrimination with a new pair. This phase of the task, the ODOR 2 set, proved much easier for the rats, who satisfied the criterion within 1.7 ± 0.4 days, supporting the hypothesis that the animals indeed learned to assign relevance to the correct sensory dimension. Finally we performed an extra-dimensional shift, switching the cue for finding the reward from an odor to a LED color. As expected, the LED set was substantially more difficult for the animals, requiring 12.6 ± 0.9 days to satisfy the same criterion. Three animals failed to reach the required success rate during this set.

The group trained on the LED-first version (Figure 2B) reached the 75% success rate during the first (LED) training set within 8.5 ± 1.5 days. following successful learning, we performed an extra-dimensional shift, introducing new stimuli and switching the reward rule to an odor-based one. In this (ODOR 1) set 9.6 ± 1.4 days were required to satisfy the successful learning criterion. One rat failed to satisfy the criterion.

### 2.2. Single trial analysis of choice behavior

Trial-to-trial analysis of the animals' choices allows us to explore learning strategies of the rats and deduce their common features.

**Figure 3:**
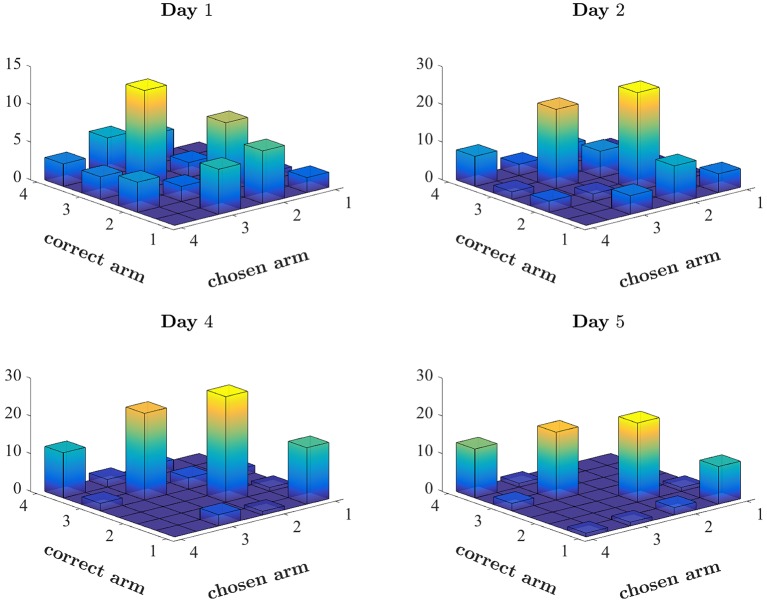
Pattern of choices made by a single rat. Number of times the rat chose each arm, plotted against the correct arm in the trial. Correctly performed choices appear on the diagonal. Spatial bias appears as disproportionate number of choices of a single arm (see for example the choices of arm 3 on Day 1). The first 2 days and the last 2 days of the first set of this rat (ODOR 1) are depicted.

For example, a rat may follow a spatial strategy, searching for an arm associated with the highest probability of success. Figure 3 depicts the arms chosen by one rat on four training days of the first set (ODOR 1) in the odor-first group. The beginning of training was typically characterized by spatial biases. Figure 3 (top left) depicts the first day of training of a single rat, in which the animal tended to avoid arm 1 and disproportionately chose arms 2 and 3. This pattern of choice was reinforced by successes associated with these arms. The rat's avoidance of arm 1 also prevented positive reinforcement related to this arm. This behavior is consistent with learning values assigned to each arm, where values express the degree of success associated with each location. However, it is not clear whether such values are assigned to every feature (location, type of odor, the color of the LED) independently or to every combination of features. Inspection of the later days of the same set, depicted in the lower panels, reveals that although the bias persists throughout training, it is less pronounced at the end, leading to a sufficiently high rate of success on the last day.

To systematically analyse of the trial-to-trial behavior, we formulated two competing models of learning and decision-making and estimated the parameters of each to fit the actual behavior of the rats. The models are presented formally in Materials and Methods, sections 4.3 and 4.4.

The first model, Naive Reinforcement Learning model (NRL) (Niv et al., 2015), described in section 4.3, is an adaptation of a classical on-line reinforcement learning procedure (Wagner and Rescorla, 1972; Sutton and Barto, 1998). In this model, every *combination* of features (e.g., the western arm delivering an apple odor and a blue LED) has a value that is recursively updated every time it is chosen, according to the current outcome (success or failure). The updated value is a weighted average between the cached value and the current outcome, and the relative weight of the latter is the learning rate α, ranging between 0 and 1, see Equation (1) in section 4.3. The policy chooses the alternative with the highest learned value with a higher probability, as determined by the softmax function. The degree of randomness in the decision is determined by the inverse temperature β ≥ 0. Lower inverse temperature corresponds to more random,“exploratory,” decisions (see Equation 2).

The Weighted Attention Model (WAM, described in section 4.4), assumes that every dimension is reinforced *separately*, i.e., a value is independently associated with each sensory input. Each of the values of a chosen stimulus is updated after every choice. However, in contrast to the NRL model, the decision is based on a *weighted average of the values* associated with sensory dimensions (location, odor, LED color). These relative weights are additional parameters of the model. As in case of NRL, the WAM also has the learning rate and inverse temperature as model parameters. The parameter estimation procedure for both models is described in detail in section 4.5.

### 2.3. Comparison of the NRL and the WAM in explaining animals' behavior

We used the two models to explain behavior of 8 rats performing the odor-first version of the task and 10 rats performing the LED-first task.

To compare the predictive power of both models, we relied on two methods, the Akaike Information Criterion (AIC) (Akaike, 1992) and cross validation. AIC comparison found that in 20/24 and 13/20 rat × set combinations in the odor-first and LED-first groups, respectively, the WAM outperformed NRL. To perform the cross-validation test, we used the first 90% of trials in each day as a training set, and the last 10% as a test set. This procedure was carried out for the NRL and the WAM. Overall, the proportions of trials explained by each model varied between 0.33 and 1. In 139 out of 166 days, the WAM predicted at least as large a fraction of test trials as the NRL. Note that both models used the beginning of each day as a training set and the end of the day trials as the test set, and naturally most of the rats perform better in the final trials than at the beginning of the day. Thus, in constructing this cross-validation we stacked the deck in favor of the NRL model, which predicts correct choices after the first success, and has no way to account for mistakes. Hence the better performance of the WAM in this case indicates that this model explains the behavior better and does not overfit, despite having more parameters.

#### 2.3.1. Mistakes are captured by the WAM better than the NRL

**Figure 4:**
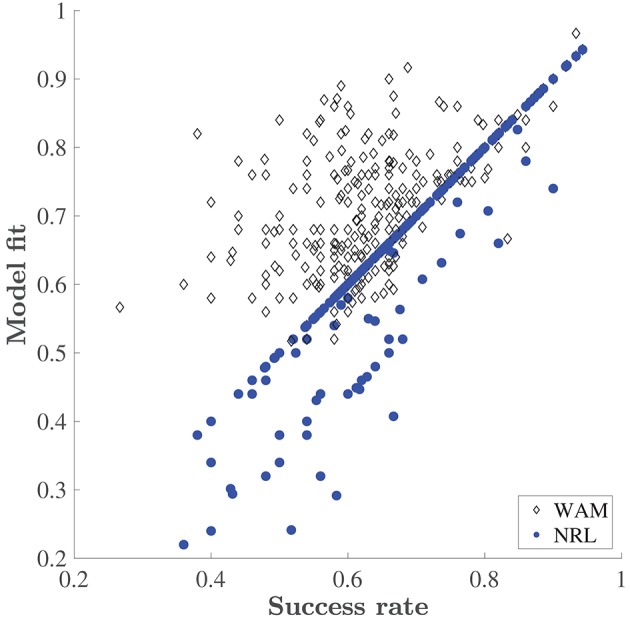
Explanatory power of the WAM vs. the NRL. Success rate is the fraction of correct choices made by a rat in a training day. Model fit is the fraction of choices correctly predicted by the corresponding model (the WAM and the NRL) without adding exploration, i.e., based on the accumulated values only.

The NRL underperforms the WAM, especially at the initial stages of learning of each new set, where the WAM captures erroneous choices which the NRL cannot. Recall, that in our task design the reward schedule is deterministic, and there are no changes in reward contingencies. Therefore, in the NRL framework, no negative prediction errors are encountered. The values associated with any stimulus combination that contains the rewarded stimulus (such as a particular odor in the ODOR 1 set or a particular color in the LEDs set) should increase with every correct choice, while all other values should not change and remain at the initial value, 0. Thus, after the first correct choice, the NRL prediction places a higher probability on a correct choice. So, in this case, without any noise or exploration the NRL predicts that only correct choices will be made. On the other hand, WAM is capable of predicting an erroneous choice (even without exploration) if a particular feature was previously associated with success. Figure 4 depicts the deterministic (no exploration) fit of both models as a function of the animals' performance. The fit of the NRL often equals the fraction of correct choices made by a rat, see the concentration of the dots on the diagonal. By contrast WAM explains mistakes, as is evident by the concentration of the WAM points above the diagonal. An increase in erroneous choices is captured in the NRL by decreasing the inverse temperature, adding “exploration”, i.e., a random deviation from the choice associated with a higher value. However, estimating the magnitude of this parameter cannot trace any systematic “pattern of mistakes” or learning dynamics. It is this problem that the WAM is designed to address.

Using the WAM we can explore an experience-driven bias in decisions by observing the dynamics of the estimated weights. To stress, in constructing the WAM we did not assume a-priori any particular algorithm for changing the weights in response to experience, i.e., we did not impose a way to explore the feature-dimension space. Rather, we let the observations determine the weights that best explain the learning behavior of the rats. As a result, we obtain estimated dynamics of the underlying learning process, which points to a possible explanation of the delay in learning after the extra-dimensional set shift.

### 2.4. Weight shifting following set-shifts

**Figure 5:**
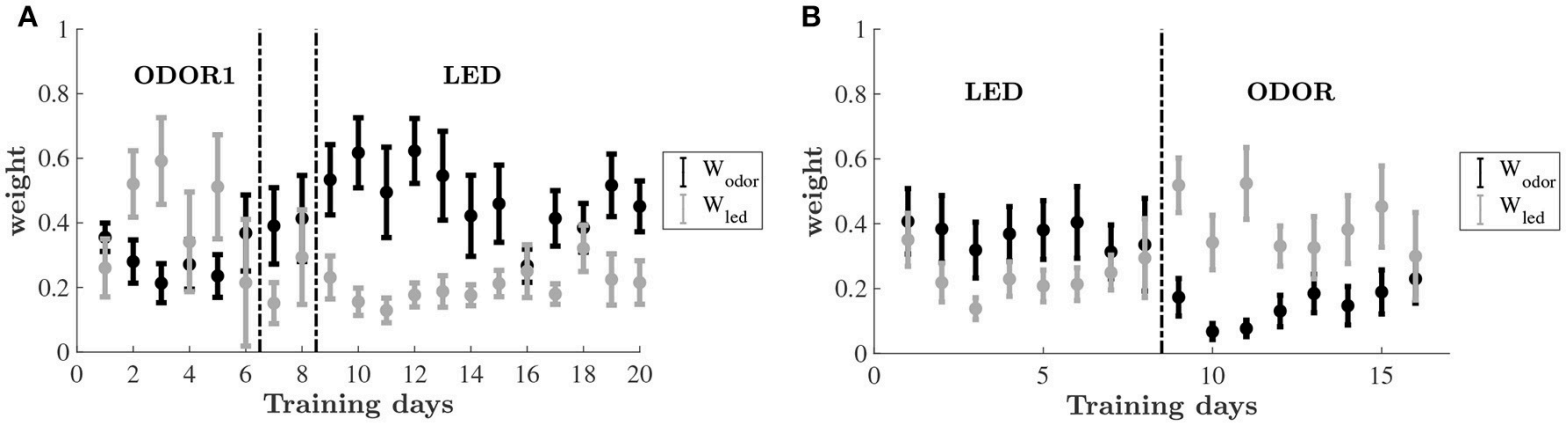
Estimated decision weights on odor and LED color by training days. Average by animal ±*SEM*. The sets are the same as in Figure 2. **(A)** Relative decision weights on the odor (black) and LED color (gray) dimensions on the odor-first group. **(B)** Relative decision weights on the odor (black) and LED color (gray) dimensions on the LED-first group.

To uncover the learning dynamics we compare the estimated decision weights associated with two different sensory dimensions: olfactory (odor) and visual (LED color). Figure 5 shows the weights associated with the two dimensions throughout the training on the odor-first and leds-first versions of the task. The dynamics of the weight allocation reveal a pattern of an emerging bias. For the group of animals that were trained on the odor-first version of the task (Figure 5A), in the ODOR 1 set we find that even as the animals improved, the weights on the relevant feature dimension, odor, did not change significantly, and did not differ from each other. However, when an intra-dimensional shift was introduced, an attentional bias appeared, and the weights on odors were higher than those on LED color.

After the extra-dimensional shift, the decision weight on odors remained higher than that of the LED colors for the early stage of learning, mirroring the lack of behavioral improvement. As animals improve this difference decreases (although never reversed).

A similar bias emerged for the led-first group, cf. Figure 5B. While in the first set nearly equal weights are assigned to odor and LED color, upon shifting to the ODOR set the pattern has changed. Here, the weight on the previously relevant dimension—the LED color—is substantially higher than the non-relevant odor. The weights converge again by the end of this phase.

A two-way repeated-measures ANOVA was carried out for testing changes in the average weights of each set during training, using phase (ODOR 1, ODOR 2, and LED) and dimension (odor, LED) as independent variables. A significant main effect was found for dimension [*F*_(1, 7)_ = 9.325, *P* < 0.05], and an interaction effect for set by dimension [*F*_(2, 14)_ = 7.836, *P* = 0.005]. A similar analysis was performed for the LED-first group, yielding a significant interaction effect for stimulus by phase [*F*_1, 7_ = 8.174, *P* < 0.05].

To examine the significance of the temporal changes in estimated decision weights throughout learning, while avoiding biases related to different duration of each set, we divided each training phase into early and late stages based on each animal's performance, and examined the weights as explained by set (ODOR 1/ODOR 2/LED), learning stage (early/late) and dimension (odor/led) (see section Materials and Methods). Three way ANOVA of the odor-first group revealed a main effect of dimension (*P* < 0.001), and a significant three way interaction (*P* < 0.0001), where the weight on odor and the weight on LED were significantly different in the early and late stages of the ODOR 2 set and the LED set (*P* < 0.0001, *P* < 0.0001 and *P* < 0.001, respectively, after bonferroni correction). In the LED-first group, no significant main effects were obtained (*P* > 0.16 for all), and the only effect was a three way interaction (*P* < 0.05). The weights on odor and on LED differed only in the early stage of the ODOR 1 set (after the extradimensional shift) (*P* < 0.001, corrected).

## 3. Discussion

Animals and humans are commonly confronted with complex situations, in which the most appropriate response is determined by signals that are often of a multidimensional nature. Identifying key features determining payoff, or even the important category (dimension) in each situation relies both on experience and on the underlying model of the world. In the series of experiments described here the dimensions are well defined: location, odor and LED color. Moreover, the reward schedule is deterministic. Therefore the task of the learner is 2-fold: to identify the relevant dimension and find the feature associated with reward within this dimension. The weighted reinforcement model introduced here allows to track the values associated with each feature independently of others and then use the weighted average of these values to form a decision.

Trial by trial behavior analysis in a multidimensional task offers several insights. In particular, examining the pattern of incorrect choices made by the animals may uncover the underlying nature of their learning and decision process. The WAM separates learning from decision-making: learning is driven by strengthening or weakening connections between the stimuli and reward, whereas decision-making is based on a weighted sum of learned associations. This dissociation implies two different sources of mistakes. Erroneous choices can be due to unsuccessful learning. Alternatively, it may be that the decision process is faulty, assigning incorrect weights to different feature-dimensions of the problem. Indeed, by separating these two behavioral components, the WAM outperformed the NRL model in explaining the trial-by-trial behavior. Whenever $\Delta V_{t}^{a,b}>0$, the deterministic NRL model predicts that the rat will choose *a*. As the animal experiences rewards, if α > 0, the value of combinations that contain the rewarded feature increases and all other values remain zero. It follows that asymptotically (as *t* → ∞), if *a* is the correction option, $\Delta V_{t}^{a,b}$ monotonically converges to 1. Thus, it is not surprising that after all combinations have been encountered at least once the proportion of correct predictions generated by this model, or the *deterministic fit*, should coincide with the success rate: only successes can be predicted but not erroneous choices, cf. Figure 4.

In the final learning set, after the extra-dimensional shift, the weights associated with the relevant feature (LED color in the odor-first group, odor in the LED-first group) were consistently lower than in previous sets, across animals. Inspection of the dynamics of weights throughout learning revealed that for most animals in the odor-first group the first days of training on the LEDs set was dominated by a high weight on odor. This weight was reflected in the poor performance and slow improvement displayed by these animals. The LED-first group displayed a mirror image of this pattern. Recall that in our model, the WAM, the third estimated weight is on location, which is not depicted in the Figure 5. Thus, the dynamics of the two depicted weights is a-priori unrestricted, they can both rise, fall, or move in opposite directions. Further, the model is rich enough to offer several ways to explain the mistaken choices—to wit, a low success rate at the beginning of the last stage might be captured by either lower inverse temperature throughout the set indicating more exploration or higher weight on either of the two irrelevant dimensions: location and LED or odor. Hence the higher estimated weight on a particular irrelevant dimension indicates that the best explanation for mistakes is a systematic reliance on an irrelevant feature as a cue. According to our results this feature belongs to the stimulus previously learned to be important for finding the reward. Thus, this result may hint at a mechanism underlying the Einstellung effect (Luchins and Luchins, 1959), in which humans after learning a task exhibit a long latent period before successfully learning a second one.

However, the weights on the relevant feature remained low, even as animals successfully learned to perform the LEDs set of the task. Therefore, the low overall weight assigned to the relevant dimension in the LEDs set cannot be attributed solely to the perseveration errors. Rather, it may be a result of the frequent change of rules that the animals faced. In a complicated, dynamic world with changing rules it might be beneficial for the animal to divide its attention and devote substantial resources to seemingly irrelevant feature-dimensions. Indeed, it has been recently shown that in a variety of tasks humans continuously search for patterns, even as this search becomes increasingly difficult by providing feedback only on chosen actions, as in our model (Plonsky and Erev, 2017).

In a recent paper, Leong et al. find that throughout learning attention is biased toward the relevant category (Leong et al., 2017). Similar to the approach taken in that study, our decision making model involves comparing weighted scores of available options, where each dimension of a problem is associated with its weight, which in turn, is a-priori independent of the value learned for different features. Thus we, too, separate the process of associating features with outcome (reward) from the process of deciding which aspects (dimensions) of the problem to take into account when comparing available options. However, we do not use observable data to measure the weights, interpreted as allocation of attention, as in Leong et al. (2017), rather, we use the model to identify the weights given the behavioral data only. In other words, our weights are the best ones that explain the behavior, within the model we suggest. The advantage of our approach is that we do not need to rely on a particular observable data. However, we cannot estimate the weights trial-by-trial, instead we fit them day-by-day (for each animal). Despite the variability of the estimates, some visible patterns emerged, as discussed above, and those are quite different from Leong et al. (2017).

In summary, the superior performance of the WAM in explaining trial-by-trial behavior seems to point to a general principle of reinforcement learning in multidimensional environments: rather than associating a state (value) with a combination of features, learning may occur separately along several dimensions. The caveat however is that such conclusion could be a result of our task design. Whether the same principle holds for learning other classes of tasks is left for future research.

## 4. Materials and methods

### 4.1. Animals

18 male Long-Evans rats 8–10 weeks old at the beginning of the experiment were used in this study. The rats were maintained in an inverted 12 h light/12 h dark cycle with unlimited access to food. During training days water consumption was restricted to 1/2 h every 24 h. Animal weight and well-being were monitored continuously. All experimental procedures were conducted in strict accordance with Institutional Animal Care and Use Committee of the Haifa University (*Ethics approval No 334/14*), as well as the EU and NIH rules and regulations for the use of animals in science research. The animals underwent surgery for implantation of tetrodes for electrophysiological recordings either in their dorsomedial or dorsolateral striatum or in hippocampal area CA1. Results from these recordings are not reported here.

### 4.2. Apparatus and behavioral task

The apparatus used for the experimental tasks was a black plexiglass plus-shaped maze with four 10 × 60 cm arms and a 30 × 30 cm central hub, situated 1 m above the floor. The entrances to the arms were blocked by retractable automatic doors. Infra-red (IR) sensors at both ends of the arm marked times of arm entry and track completion. The central arm was equipped with 4 nose-poke apparati located on the floor 3 cm away from each door. The nose poke devices had IR sensors to detect nose entry, 3 colored LEDs (blue, yellow, and green), and two tubes through which odorised air was delivered and pumped out. Each odor delivery tube could deliver 2 different odors. The odors used were commercial odors that are regularly used in the cosmetics and food industry, diluted 1:1,000 from commercial concentrated liquid. Each arm was marked with different large visible cues along the walls. Experiments were conducted in dim light. Each trial started with the rats located in the central hub of the maze. After a variable inter-trial-interval, a buzz was sounded and 2 pseudo-randomly chosen nose pokes were illuminated by LEDs of different colors. Two different odors were delivered to the same nose-pokes and pumped out to keep the odors localized to the nose-poke device. The animals initiated a trial by poking their noses into one of the two nose pokes (or both). Upon this, the doors of the corresponding two arms opened and allowed access to the arms. the animals could now enter either one of the open arms. If the correct arm was chosen and traversed within the allowed maximum time of 5 seconds, the rats were rewarded by 0.3 ml of water delivered at a reward port at the end of the arm. The correct arm depended on the experimental condition. In the odor sets of the experiment, reward was delivered in the arm associated with a specific odor (and a random LED) and for the LED set, reward was delivered in the arm associated with a specific LED (and a random odor). Rats were trained for 50–100 daily trials until they satisfied the criterion performance of 75% correctly performed trials. We assigned the animals into two groups. One group of animals was trained on the odor-first version of the task, and the second group on the LED-first version. In the odor-first group, animals were first trained an an odor set (ODOR 1). Following successful learning, we performed an intra-dimensional shift, in which a new pair of odors was delivered, and the animals had to associate one the new odors with reward. When animals reached the threshold performance for this pair as well they underwent an extra-dimensional shift, in which they had to learn to associate the reward with one of the two possible LED colors, disregarding odors. The extra-dimensional shift was also accompanied with introduction of a new pair of odors. The LED-first group was first trained on a LEDs set, and after successful learning underwent an extradimsional shift and had to follow an odor rule (with a new pair of odors). We did not perform an intra-dimensional shift with this group.

### 4.3. Naive reinforcement learning model

#### 4.3.1. Values

As a first approximation, we look for the best fit of the “naïve reinforcement learning” model (NRL) (Niv et al., 2015) to the behavioral data. Every combination of observable features (e.g., arm 3, green LED and apple odor) is defined as a “state” whose value is to be learned. Thus, if at trial *t* an arm *i* with odor *j* and LED color *k* is chosen, the corresponding value *V*_*i,j,k, t*_ is updated. Update follows the reinforcement learning (RL) rule,

(1) $$\begin{array}{r} V_{i,j,k,t}-V_{i,j,k,t-1}=\alpha \left(R_{t}-V_{i,j,k,t-1}\right) \end{array}$$

where α ∈ [0, 1] is the learning rate, *R*_*t*_ = 1 if reward was received and *R*_*t*_ = 0 otherwise. Initial values *V*_*i,j,k, t* = 0_ are set to zero. Values corresponding to non-chosen options are not updated. Hence by construction all the values are between zero and one. Updated values for each feature combination were carried over between consecutive days of each learning set (see detailed description in section *Model estimation procedure*).

To capture the dynamic nature of the learning process, we allowed α to change daily. Parameter estimation was performed by log-likelihood maximization, as explained in section *Model Estimation Procedure*.

#### 4.3.2. Decision rule

Decisions are based on the values associated with each available option. Initial values are set equal to zero, as is mentioned above. At every trial *t* we calculate the difference between learned values $\Delta V_{t}^{a,b}$ of the two available choices, *a* and *b*. A deterministic decision rule states that the predicted choice will follow the option with the higher value.

The stochastic nature of the animals' choices is captured by the softmax function, which describes the probability of choosing an alternative as a function of its relative value. The probability of choosing option *a* at trial *t* is

(2) $$\begin{array}{r} P_{t}=\frac{1}{1+e^{-\beta \Delta V_{t}^{a,b}}} \end{array}$$

The inverse temperature parameter β ≥ 0 is held constant for all days in a given set and is estimated along with α using maximum likelihood to fit the data, as explained in section *Model Estimation Procedure*.

### 4.4. The weighted attention model

To capture the animals' learning and choice behavior better, we devised a modified reinforcement learning model which incorporates feature-by-feature reinforcement with a decision process that employs differential attention to distinct feature dimensions, the weighted attention model (WAM).

The model generates a prediction of the choices made by a rat in each trial. The two basic components of the model are the *values*, which are computed separately for each alternative, stored in memory and updated after each trial according to the *learning rule* below, see Equations (3–5), and the *decision rule*, see Equation (6).

#### 4.4.1. Values

In any given trial, action values have 3 components reflecting the three feature dimensions: location, odor and LED color. By design, only one of these components was relevant for getting the reward. Location was never relevant in these experiments (unbeknownst to the rats).

The location component contains 4 variables indicating the values for each of the four arms, cf. Figure 1. Denote the values at trial *t* by (*l*_1*t*_, *l*_2*t*_, *l*_3*t*_, *l*_4*t*_).

The odor component is a pair, containing the values for each of the two distinct odors: (*o*_1*t*_, *o*_2*t*_). In the odor set, we let *o*_1*t*_ be the value corresponding to the *correct odor*, which is the key to finding the reward, whereas *o*_2*t*_ be the value of the second odor, *incorrect odor*, never associated with the reward.

The LED color component at trial *t* is also a pair, (*c*_1*t*_, *c*_2*t*_). Likewise, in the LED set the first value is associated with the *correct* LED color.

At the beginning of the series of experiments (*t* = 0) all the values are set to zero.

Values are updated on each trial *t* after the rat has completed its choice and received the feedback (reward/no reward). Assume at trial *t* the animal chose location *i* with odor *j* and LED color *k*. Then the following three variables are updated according to the same reinforcement learning (RL) rule:

(3) $$\begin{array}{r} l_{it}-l_{i,t-1}=\alpha \left(R_{t}-l_{i,t-1}\right) \end{array}$$

(4) $$\begin{array}{r} o_{jt}-o_{j,t-1}=\alpha \left(R_{t}-o_{j,t-1}\right) \end{array}$$

(5) $$\begin{array}{r} c_{kt}-c_{k,t-1}=\alpha \left(R_{t}-c_{k,t-1}\right) \end{array}$$

where learning α is the learning rate, and *R*_*t*_ is the reward in trial *t*, which is 1 in case of success and 0 in case of failure. The values of all the unchosen features in a given trial are not updated.

#### 4.4.2. Decision rule

The decision rule can be affected by the values corresponding to the three observable dimensions of each available alternative: location, odor and color. Each value affects the decision through a corresponding relative weight, *w*_*l*_, *w*_*o*_, *w*_*c*_. All the weights are positive and sum up to 1.

A choice of arm *i*, with odor *j* and LED color *k* receives the composite value score

$$\begin{array}{l} w_{l}l_{it}+w_{o}o_{jt}+w_{c}c_{kt} \end{array}$$

Choices are made by comparison of the composite value scores, and their difference is the decision index. Let option *a* be arm *i* with odor *j* and LED color *k* and the other available option be *b* with arm *i*′, odor *j*′ and LED color *k*′, then the decision index is

(6) $$\begin{array}{r} I_{t}^{a,b}=w_{l}\left(l_{it}-l_{i^{\prime }t}\right)+w_{o}\left(o_{jt}-o_{j^{\prime }t}\right)+w_{c}\left(c_{kt}-c_{k^{\prime }t}\right) \end{array}$$

The deterministic model predicts the choice *a* if $I_{t}^{a,b}>0$ and *b* otherwise.

In the stochastic decision rule, the predicted probability of a choice depends on the relative weighted decision index according to the softmax function, determined by the inverse temperature parameter β, as in the NRL, thus the probability of choosing *a* is *I*_*t*_ is

(7) $$\begin{array}{r} P_{t}=\frac{1}{1+e^{-\beta I_{t}^{a,b}}} \end{array}$$

### 4.5. Model estimation procedure

The parameters of the NRL model, the learning rate and the inverse temperature, {α_*i*_, β}, for every day *i* of a given set, were estimated separately for each set of the experiment. The learning rate is restricted to be 0 ≤ α_*i*_ ≤ 1 and inverse temperature is restricted to be 0 ≤ β ≤ 30.

Within every learning set the values for each combination of features are updated trial-by-trial (according to Equation 1) and carried over to the following day. These values are set to zero at the beginning of each set. Parameter estimation is performed by log-likelihood maximization. The maximization was implemented using a built-in constrained optimization routine in Matlab with the initial guess being the best parameter combination found using the simple search over a thin grid of possible parameters.

The parameters of the WAM are the relative weights and the learning rate for every day *i* of a given set: {*w*_*i*_ = (*w*_*li*_, *w*_*oi*_, *w*_*ci*_), α_*i*_}, where the weights 0 ≤ *w*_*li*_, *w*_*oi*_, *w*_*ci*_ ≤ 1 sum to 1, α_*i*_ ∈ [0, 1]. In addition, we estimate the inverse temperature parameter 0 ≤ β ≤ 30, which is fixed throughout the set. Hence there are 3 free parameters for every day of the experiment, and one parameter for every set.

The parameters are estimated separately for every set of the experiment, as for the previous model. Within every learning set the values associated with each feature are updated trial-by-trial (according to Equation 3–5) and carried over to the following day. These values are set to zero at the beginning of each set. The estimation procedure otherwise is similar to that of the NRL model.

Parameter estimates in both cases are robust to small decay between days, i.e., carrying over to the next day a discounted array of values.

### 4.6. Model comparison

We used two methods to compare the explanatory power of the NRL model and WAM. First, we used the Akaike Information Criterion (AIC), (Akaike, 1992), calculated as follows:

(8) $$\begin{array}{r} \ln\;L_{WAM}-\ln\;L_{NRL}-d \end{array}$$

where *L*_*WAM*_, *L*_*NRL*_ are the likelihood of the weighted attention and naive reinforcement learning models respectively, *N* is the number of days in a given set and *d* = 2 × *N* is the difference in the number of parameters in the two models. Only days in which the fit of NRL was over 50% were used for comparison. We further performed a cross-validation analysis, where we used the first 90% of the trials in each day as a training set to estimate parameters and examined it on the remaining 10% of the trials. The obtained fits to the data were compared between models.

### 4.7. Statistical analysis

For statistically verifying the demonstrated changes in calculated weights across training sets and weight dimensions, we carried out two-way repeated measures ANOVA tests on the average weights of each set (before and after each shift) and dimension (odor, LED), throughout training. To further compare the weights on odor and LED in each of the phases we divided each learning phase into an early and late stage according to animal performance. We determined a threshold of 70% for each animal as the first day of the late stage. For each of the 8 animals in the odor-first group the weights were characterized by 2 dimensions (ODOR and LED), 3 sets (ODOR1, ODOR2, LED) and two stages (early, late). For each of the 10 animals in the LED-first group there were also 2 dimensions and 2 stages but only 2 sets and compared with a 3 way ANOVA.

## Author contributions

GM designed the experiments. FA performed the experiments. AR designed the model. FA, AR, and GM analyzed the data. AR and GM wrote the manuscript.

### Conflict of interest statement

The authors declare that the research was conducted in the absence of any commercial or financial relationships that could be construed as a potential conflict of interest.
